# An Efficient Numerical Homogenization Method for Multi-Scale Modeling of 2.5D Package Warpage and Thermal Analysis

**DOI:** 10.3390/mi17070853

**Published:** 2026-07-17

**Authors:** Pengying Xu, Shaoyi Liu, Lu Hao, Jitang Zhang, Yan Wang, Qiulin Tan, Congsi Wang

**Affiliations:** 1 Science and Technology on Electronic Test and Measurement Laboratory, North University of China, Taiyuan 030051, China; xupengying1993@163.com (P.X.); zhangjitang2006@163.com (J.Z.); tanqiulin@nuc.edu.cn (Q.T.); 2 State Key Laboratory of Extreme Environment Optoelectronic Dynamic Measurement Technology and Instrument, North University of China, Taiyuan 030051, China; 3 Beijing Institute of Aerospace Microsystem and Information Technology, Beijing 100094, China; 4 College of Information and Control Engineering, Xi’an University of Architecture and Technology, Xi’an 710055, China; wangyan5169@163.com; 5 State Key Laboratory of Electromechanical Integrated Manufacturing of High-Performance Electronic Equipments, Xidian University, Xi’an 710071, China; congsiwang@163.com

**Keywords:** numerical homogenization, multi-scale analysis, 2.5D packaging, periodic boundary condition, warpage analysis, thermal analysis

## Abstract

To achieve high interconnect density in 2.5D packages, various microscale structures such as through-silicon vias (TSVs), microbumps, and redistribution layers (RDLs) are employed. These features typically exist at the micron scale, whereas other package components span millimeter to centimeter scales, resulting in a wide range of physical dimensions within the package. Although finite element analysis (FEA) has proven effective for evaluating the mechanical and thermal characteristics of 2.5D packages, the inherent multi-scale nature poses significant computational challenges and numerical convergence issues, severely hindering the design and analysis of increasingly dense packages. To address this problem, this paper proposes an efficient numerical homogenization method for the mechanical and thermal analysis of 2.5D packages. The method employs periodic boundary conditions (PBCs) based on the concept of referential statistical volume elements (rSVEs). In this approach, typical microstructures—including TSVs, microbumps, and RDL traces together with the surrounding matrix material—are treated as a homogeneous medium, and the equivalent material properties of the multi-scale structures are evaluated. These properties include the stiffness matrices (from which the equivalent Young’s modulus, shear modulus, and Poisson’s ratio can be derived), coefficients of thermal expansion, and thermal conductivity. Validation results demonstrate that the proposed method ensures continuity of displacement, stress, strain, and heat flux across opposite surface pairs of the rSVEs. Compared with experimental measurements and other existing homogenization techniques, the method accurately determines the equivalent material properties of complex multi-scale structures without being restricted to specific geometries, while significantly improving computational efficiency. Finally, the proposed numerical homogenization method is successfully applied to wafer warpage analysis during the manufacturing process and to thermal analysis under operating conditions. The results indicate that the method achieves high computational efficiency while maintaining accuracy in both mechanical and thermal analyses of 2.5D packages, thereby laying a solid foundation for the development of next-generation 2.5D package structures.

## 1. Introduction

As the integration density of modern electronic chips continues to increase and transistor counts rise, the demand for higher interconnect density within packages has grown rapidly. However, as Moore’s law approaches its fundamental physical limits, achieving further density improvements through transistor scaling alone is becoming increasingly difficult. Consequently, 2.5D integrated circuits (ICs) have attracted significant attention as an effective means to improve overall chip system performance [1]. In this approach, 2.5D packaging not only provides mechanical protection and electrical interconnection—functions traditionally associated with conventional 2D packaging—but also facilitates shorter interconnects, system architecture reconfiguration, and higher functional block density. To achieve these advantages, microbumps, TSVs, and RDLs are widely integrated into 2.5D package structures. However, the incorporation of these microscale interconnect features introduces feature sizes spanning several orders of magnitude, from micrometers to millimeters or even centimeters.

Computer-aided engineering (CAE) technologies, which enable virtual manufacturing and performance validation during the early design stage, are crucial for reducing development costs and improving product yield [2]. In particular, FEA is extensively used to evaluate the thermomechanical responses of 2.5D packages during fabrication and under operating conditions [3,4]. Nevertheless, the inherent multi-scale nature of 2.5D packages makes FEA modeling extremely challenging: fine meshes are required for each component to accurately resolve displacement and strain distributions, often leading to an unmanageable number of elements. This results in prohibitive computational time and numerical convergence difficulties, even when substantial computational resources are available [5,6].

To address the multi-scale challenges inherent in 2.5D packages, homogenizing the micro-interconnect structures together with the surrounding matrix material and treating them as an equivalent homogeneous medium is an effective analytical strategy. By first determining the effective material properties of the equivalent homogeneous structure and then performing FEA on this homogenized representation, the numerical convergence can be significantly accelerated. Various homogenization methods have been reported, which can be broadly classified into three categories: (a) experimental measurements [7], (b) analytical models [8], and (c) numerical simulations [5,9]. Experimental measurements provide the most reliable assessments of material properties, yet they are prohibitively expensive and time-consuming. More importantly, they can only be performed after fabrication, rendering them impractical for early-stage design optimization. Analytical models can accelerate the computational process; however, they become cumbersome—and often infeasible—for complex package structures. Furthermore, analytical models typically rely on oversimplifying assumptions [10] regarding mechanical field variables or the geometric features of microstructures, which yield only approximate estimates of the equivalent properties. In contrast, numerical methods are capable of accommodating the intricate multi-scale architectures of 2.5D packages more effectively, offering more accurate predictions of equivalent material properties along with detailed insights into the microscopic fields within the multi-scale structures. As a result, numerical homogenization has emerged as a robust and versatile approach for determining the equivalent properties of multi-scale structures in 2.5D packages.

The concept of the representative volume element (RVE)—a unit cell whose response statistically represents that of a heterogeneous medium—has evolved into a cornerstone of numerical homogenization. In this approach, the effective properties of the unit cell are determined via FEA, thereby circumventing exhaustive multi-scale analyses. However, when applying the finite element method to extract equivalent material properties for 2.5D package multi-scale structures, meticulous attention must be paid to the implementation of boundary conditions (BCs). Different BCs yield divergent effective properties, and inappropriate constraints may introduce non-physical artifacts, leading to entirely erroneous outcomes [11]. Furthermore, satisfying the Hill–Mandel condition [12] is a prerequisite for the rigorous derivation of equivalent properties. While kinematic uniform boundary conditions (KUBCs) and static uniform boundary conditions (SUBCs) are prevalent in existing RVE-based studies, prior research indicates that KUBCs systematically underestimate mechanical properties, whereas SUBCs induce the opposite bias [13]. Periodic boundary conditions (PBCs) [14], which inherently satisfy the Hill–Mandel energy principle, are deemed more suitable for numerical homogenization and, if properly implemented, accurately capture the equivalent properties of 2.5D package architectures. Despite these advantages, PBC applications to such structures remain scarce. Moreover, characterizing these structures necessitates simultaneous evaluation of multiple parameters: Young’s modulus, shear modulus, Poisson’s ratio, the coefficient of thermal expansion (CTE), and thermal conductivity. Although PBCs have been deployed to address isolated mechanical or thermal phenomena in other domains [10,15], an integrated computational framework capable of concurrently determining these multifaceted properties remains absent.

**Figure 1:**
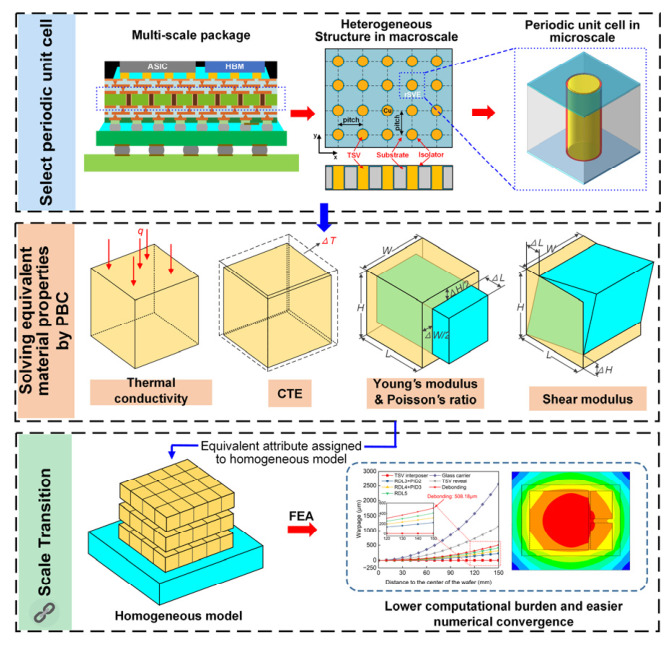
The framework of multi-scale analysis based on the numerical homogenization.

To address these gaps, the principal advances of this work are threefold. First, we establish a unified PBC-based numerical homogenization framework that integrates the determination of the stiffness matrix, CTE, and thermal conductivity within a single algorithmic workflow specifically tailored to the multi-scale architectures of 2.5D packages. Second, we provide detailed prescriptive guidelines—including explicit boundary condition application rules in tabular form—that serve as a practical reference for engineers performing homogenization of package structures. Third, through application to wafer warpage and thermal analyses, we unveil several previously obscured engineering insights, including the critical role of the SiO_2_ liner in TSV thermal behavior, the substantial warpage suppression afforded by backside RDLs, and the finding that equivalent properties are independent of the specific rSVE morphology when constituent volume fractions are conserved. Collectively, these contributions advance the state of the art by providing a computationally tractable and physically robust homogenization paradigm for multi-scale analysis of 2.5D packages. The overall workflow of the proposed framework is illustrated in Figure 1.

The PBC implementation strategy proposed herein facilitates the determination of key equivalent properties for multi-scale 2.5D package architectures, thereby addressing the paucity of tailored numerical homogenization techniques. This work elaborates on the selection protocol for periodic unit cells pertinent to 2.5D integration and verifies the continuity of stress and strain fields at antipodal nodal pairs to ensure rigorous satisfaction of the Hill–Mandel condition. The computational efficacy and accuracy of the proposed algorithm are further benchmarked against archival datasets. The study culminates in demonstrative case studies, encompassing wafer warpage analysis during fabrication and thermal profiling under operational conditions. This work is poised to substantially accelerate structural design optimization, harnessing the full potential of CAE for virtual prototyping.

## 2. Periodic Structural Characterization of 2.5D Package

**Figure 2:**
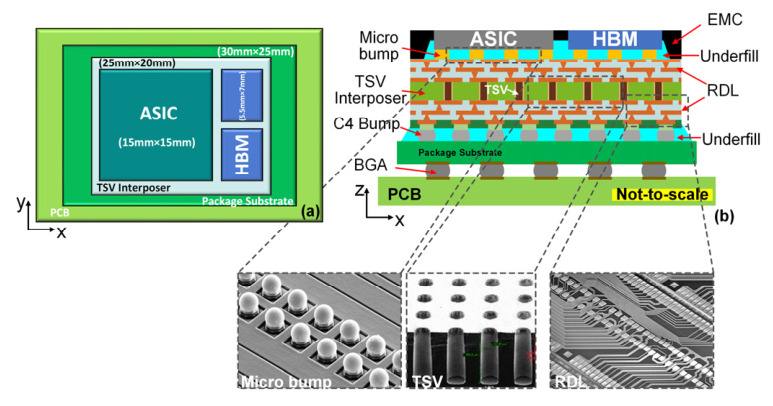
Schematics of a typical 2.5D package. (**a**) Layout of 2.5D package; (**b**) complex multi-scale structure in 2.5D package.

Figure 2 illustrates the structural characteristics of a 2.5D package, which comprises both large-scale components such as chips, substrates, and interposers, and microscale interconnect elements including microbumps, TSVs, and RDL traces. The multi-scale nature of these structures renders thermo-mechanical analysis of 2.5D packages particularly challenging.

Despite the significant scale disparity, TSV arrays are periodically embedded within the silicon substrate, microbumps are statistically homogeneous throughout the underfill material, and the Cu traces in the RDL are architecturally configured within the polyimide (PI) dielectric layer [16], as depicted in Figure 2b. Consequently, each layer of the 2.5D package can be regarded as a multiphase material system formed by the architectural configuration of materials with distinct properties at different scales. This results in a medium that, although heterogeneous at the microscale, can be treated as a homogeneous continuum at the macroscopic level for properties such as modulus, Poisson’s ratio, CTE, and thermal conductivity through the application of numerical homogenization techniques. This approach obviates the necessity of explicitly resolving the complexities of the multi-scale structures and facilitates a tractable macroscopic analysis by enabling the overall behavior of the 2.5D package to be evaluated in terms of its equivalent material properties.

**Figure 3:**
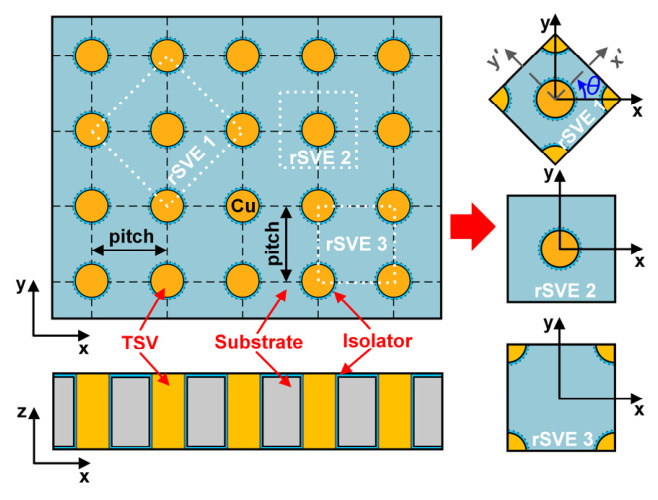
The TSV array and its rSVE models.

The first step in this work is to identify a representative region that captures all essential microstructural details and enables accurate property evaluation; this region is designated as the rSVE (referential statistical volume element). Following the conceptual framework of the study [17], the classical RVE is defined as the asymptotic limit at which apparent properties become independent of boundary conditions and statistical fluctuations vanish, whereas the SVE (Statistical Volume Element) refers to any finite mesoscale window that has not yet reached this limit. The rSVE introduced here is a specific type of SVE whose dimensions are dictated by the geometric periodicity of 2.5D package design rules (e.g., TSV pitch, RDL trace spacing), rather than by statistical convergence criteria, and it directly serves as the repeating unit for PBC-based homogenization, bypassing the need for size-convergence validation. This distinction is important because the microstructures in 2.5D packages (TSVs, microbumps, and locally periodic RDL trace blocks) are arranged in regular periodic patterns by design, making the RVE’s asymptotic convergence requirement excessively restrictive in this engineering context. Acting as a bridge between macroscopic and microscopic scales, the rSVE reflects the intrinsic characteristics of the multiphase material and reproduces its equivalent properties, thereby streamlining the thermo-mechanical analysis. Figure 3 illustrates the periodic arrangement of vias within a TSV interposer; at the macroscopic level, the interposer is formed by periodic repetition of the rSVE, which comprises Cu, SiO_2_, and Si with volume fractions identical to those in the macroscopic layer. The same principle applies to the RDL and microbump/underfill rSVEs. Furthermore, the rSVE terminology is also adopted to complement parallel investigations into globally non-periodic RDL layouts, where the concepts of local periodicity and critical partition scales will be further developed, thus providing a consistent conceptual foundation for this research trajectory.

## 3. PBC-Based Numerical Homogenization Strategy

### 3.1. Boundary Conditions for Numerical Homogenization

**Figure 4:**
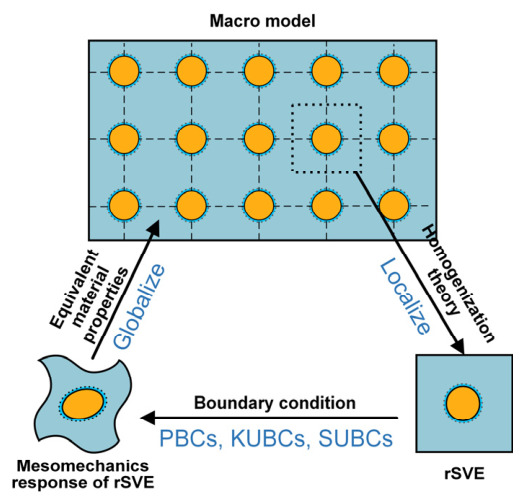
The workflow of numerical homogenization.

In the context of a 2.5D package, when considering a macroscopic heterogeneous continuous structure denoted as V that contains a representative microstructure Ω, numerical homogenization techniques are employed to address the thermo-mechanical challenges at both the macro and micro scales. As illustrated in Figure 4, the process of extracting and analyzing the microstructure from the macroscopic heterogeneous material is designated as localization. Conversely, the process of volume-averaging the properties of the microscale rSVE and incorporating them as equivalent properties into the macroscopic analysis is termed globalization. Localization enables a detailed investigation of the material’s microstructure, while globalization leverages these microscale insights to characterize the material’s macroscopic behavior. This coupled localization–globalization framework is pivotal for accurately modeling and predicting the performance of materials in 2.5D packages.

In the context of multi-scale structures in 2.5D packages, the rSVEs must be deployed in a periodic tessellation throughout the macroscopic domain, with explicit interfacial delineation relative to adjacent regions. To accurately determine the equivalent material properties through numerical homogenization, it is essential to enforce admissible boundary constraints on the selected rSVE. The boundary conditions satisfying the Hill–Mandel condition can be broadly categorized into three types: KUBCs, SUBCs, and PBCs cf. [18].

KUBC implies that the displacement $\underline{u}$ is applied to points *x* that belong to the boundary ∂Ω:

(1) $$u_{i}=E_{ij}x_{j}\;\forall \underline{x}\in \partial \Omega$$

where $E_{ij}$ represents the components of a given symmetric second-order tensor that is independent of $\underline{x}$ in the cartesian coordinate system. This implies that the average strain on Ω is:

(2) $$\langle \varepsilon_{ij}\rangle ≙\frac{1}{V}\int_{\Omega }\varepsilon_{ij}dV=E_{ij}$$

then, the macroscopic stress tensor $\sum_{ij}$ is defined by the volume average:

(3) $$\sum_{ij}≙\langle \sigma_{ij}\rangle =\frac{1}{V}\int_{\Omega }\sigma_{ij}dV$$

SUBC denotes the specification of a traction vector at the boundary:

(4) $$\sigma_{ij}n_{j}=\sum_{ij}n_{j}\;\forall \underline{x}\in \partial \Omega$$

where $\sum_{ij}$ represents the components of a given symmetric second-order tensor that is independent of $\underline{x}$. The $\underline{n}$ denotes the outward normal vector at point $\underline{x}$ on the boundary ∂Ω, therefore:

(5) $$\langle \sigma_{ij}\rangle =\frac{1}{V}\int_{\Omega }\sigma_{ij}dV=\sum_{ij}$$

then, the macroscopic strain tensor $E_{ij}$ is defined by the volumetric average:

(6) $$E_{ij}≙\langle \varepsilon_{ij}\rangle =\frac{1}{V}\int_{\Omega }\varepsilon_{ij}dV$$

The stress and strain fields within a periodic unit cell exhibit continuity and periodicity under the influence of external loads. For the PBC, the displacement field across the entire boundary ∂Ω adopts the following form:

(7) $$u_{i}=E_{ij}x_{j}+v_{i}\;\forall \underline{x}\in \partial \Omega$$

where the displacement perturbation $\underline{v}$ is periodic, taking the same value at two homologous points on opposite faces of Ω. The traction vector $\sigma_{ij}n_{j}$ takes opposite values at two homologous points on opposite faces of ∂Ω. This implies that the PBC is satisfied.

(8) $$\int_{\Omega }\sigma_{ij}v_{i,j}dV=0,\;\mathrm{and}\;\langle \varepsilon_{ij}\rangle =E_{ij}$$

the total stress tensor can be calculated from $\sum_{ij}≙\langle \sigma_{ij}\rangle =\frac{1}{V}\int_{\Omega }\sigma_{ij}dV$.

The PBC, KUBC, and SUBC all satisfy the Hill–Mandel condition, which pertains to the local and global work done by internal forces:

(9) $$\langle \sigma_{ij}:\varepsilon_{ij}\rangle =\langle \sigma_{ij}\rangle :\langle \varepsilon_{ij}\rangle =\sum_{ij}E_{ij}$$

this means that the Hill–Mandel condition is an essential expression for solving the equivalent mechanical properties based on the rSVE.

For heat conduction problems, apply similar boundary conditions on ∂Ω. The uniform gradient of temperature condition (UGTC), which is equivalent to specifying the temperature of any point $\forall \underline{x}\in \partial \Omega$ on the boundary,

(10) $$T=G_{j}x_{j}\Rightarrow \langle T_{,i}\rangle =G_{i}$$

where $G_{i}$ is the given constant temperature gradient. The total heat flux is defined as

(11) $$Q_{i}\hat{=}\langle q_{i}\rangle$$

According to the dual condition called the uniform heat flux condition (UHFC), the heat flux at any point $\forall \underline{x}\in \partial \Omega$ is given by the following equation:

(12) $$q_{i}n_{i}=Q_{i}n_{i}\Rightarrow \langle q_{i}\rangle =Q_{i}$$

where $Q_{i}$ is the macro heat flux.

The form of PBC representing the temperature field is given by the following formula:

(13) $$T=G_{i}x_{i}+t,\;\forall \underline{x}\in \Omega \Rightarrow \langle T_{,i}\rangle =G_{i}$$

where the temperature fluctuation *t* is periodic. The heat flux $q_{i}n_{i}$ is antiperiodic. Then calculate the macro heat flux using $Q_{i}\hat{=}\langle q_{i}\rangle$.

The macro energy dissipation and micro energy dissipation of these three boundary conditions all meet the following relationship:

(14) $$\langle q_{i}T_{,i}\rangle =\langle q_{i}\rangle \langle T_{,i}\rangle =Q_{i}G_{i}$$

Please note that due to the non-uniformity of micro rSVE, the stress or displacement vector on the boundary ∂Ω of rSVE fluctuates strongly and transits from one state to another. This makes it impossible for KUBC and SUBC (and, by the same token, UHFC and UGTC) to theoretically yield strictly equivalent the thermomechanical properties of the macrostructure. At the same time, it has been proved that the PBC can obtain more accurate equivalent thermodynamic properties than other BCs [10,19].

### 3.2. Numerical Execution Strategy for the Periodic Boundary Condition

In this paper, the PBC was implemented using the commercial finite element software package. For a selected rSVE, its boundaries can be divided into two parts, $\partial \Omega^{+}$ and $\partial \Omega^{-}$, and satisfy the conditions $\partial \Omega =\partial \Omega^{+}\cup \partial \Omega^{-}$ and $\emptyset =\partial \Omega^{+}\cap \partial \Omega^{-}$. $\forall {\underline{x}}^{+}\in \partial \Omega^{+}$ corresponds to a unique ${\underline{x}}^{-}\in \partial \Omega^{-}$, and the normal vectors at these boundaries satisfy the relationship **n^+^** = −**n^−^**. Therefore, after meshing, it is necessary to ensure that all opposite surface pairs of the rSVE have the same mesh to facilitate the application of the PBC. Leveraging the mirror symmetry of the rSVE boundaries, Equation (7) can be formulated as follows:

(15) $$\begin{array}{l} u_{i}^{k+}=E_{ij}^{0}x_{j}^{k+}+v_{i}\;\mathrm{on}\;\partial \Omega^{+}\\ \mathrm{and}\\ u_{i}^{k-}=E_{ij}^{0}x_{j}^{k-}+v_{i}\;\mathrm{on}\;\partial \Omega^{-} \end{array}$$

where the superscripts *k*+ and *k*− represent the *k*-th pair of opposite and parallel boundary surfaces of the rSVE. Due to the periodic nature, the displacement perturbation *v_i_* is identical at the two parallel and opposite boundaries; hence, the difference in the aforementioned equation can be expressed as:

(16) $$u_{i}^{k+}-u_{i}^{k-}=E_{ij}^{0}\left(x_{j}^{k+}-x_{j}^{k-}\right)=E_{ij}^{0}\Delta x_{j}^{k}$$

One advantage of using the aforementioned equation is that it eliminates the displacement perturbation term *v_i_*, which is typically unknown. Note that the superscript ${\left(\cdot \right)}^{0}$ denotes a prescribed value that is applied, while the $\Delta x_{i}^{k}=x_{i}^{k+}-x_{i}^{k-}$ represents the distance between each pair of nodes on the parallel boundary surfaces, edges, and corner points within the rSVE, a constant value. Consequently, the right-hand side of Equation (16) will become a constant, determined by the imposed strain $E_{ij}^{0}$ and the dimensions of the rSVE. The application of this equation ensures the continuity of the displacement field. Additionally, this equation is a special type of displacement BC that specifies the difference in displacement between two opposite boundaries, rather than providing a prescribed boundary displacement value.

For the traction continuity condition, it can be expressed by the following:

(17) $$\tau_{i}^{+}+\tau_{i}^{-}=0\;\mathrm{with}\;\tau_{i}=\sigma_{ij}^{0}n_{j}$$

where ${\hat{u}}_{i}$ represents the macroscopic displacement caused by the imposed macroscopic strain $E_{ij}^{0}$. In fact, the application of the former part of the equation ensures the uniqueness of the solution, which implies that the latter BC does not need to be explicitly applied in FEA.

**Figure 5:**
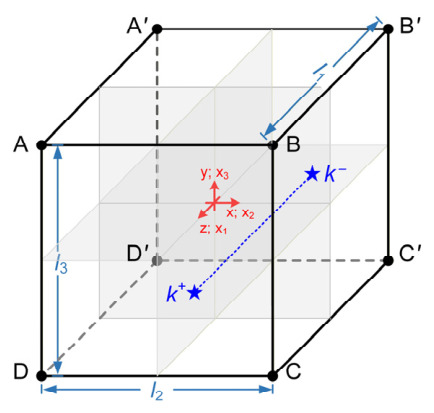
Category for the set of the nodes on the boundaries of the rSVE.

To facilitate the implementation of PBC, the planar depiction and numbering of the cubic rSVE are shown in Figure 5, with its width, length, and height denoted as *l*_1_, *l*_2,_ and *l*_3_, respectively. The coordinate system is defined as follows: axis 1 is aligned with the z-direction (vertical direction, typically the axial direction of vias in TSVs or microbumps), axis 2 corresponds to the x-direction (in-plane horizontal direction), and axis 3 corresponds to the y-direction (orthogonal in-plane horizontal direction). This convention is adopted throughout the manuscript for the stiffness matrix, CTE, and thermal conductivity tensor components. To implement PBCs, the rSVE must first be partitioned into three boundary subsets: Surface, Edge, and Corner. The details of the division are:

Surface: ABCD, A′B′C′D′, AA′B′B, DD′C′C, AA′D′D and BB′C′C;

Edge: AB, A′B′, DC, D′C′, AA′, BB′, DD′, CC′, AD, BC, A′D′ and B′C′;

Corner: A, B, C, D, A′, B′, C′, and D′.

#### 3.2.1. Equivalent Elastic Property Evaluation

To assess the overall stiffness matrix *C* of the multi-scale structure, the rSVE is subjected to an average strain ${\bar{\varepsilon }}_{\beta }$. The strain $\varepsilon_{ij}^{0}$, with its six components, is applied by enforcing the following boundary conditions on the displacement components:

Set I: Surface

1. Surface ABCD and A′B′C′D′

(18) $$u_{i}^{S-ABCD}-u_{i}^{S-A^{\prime }B^{\prime }C^{\prime }D^{\prime }}=l_{1}\varepsilon_{i1}\;\mathrm{and}\;i=1,2,3$$

2. Surface BCC′B′ and ADD′A′

(19) $$u_{i}^{S-BCC^{\prime }B^{\prime }}-u_{i}^{S-ADD^{\prime }A^{\prime }}=l_{2}\varepsilon_{i2}\;\mathrm{and}\;i=1,2,3$$

3. Surface ABB′A′ and DCC′D′

(20) $$u_{i}^{S-ABB^{\prime }A^{\prime }}-u_{i}^{S-DCC^{\prime }D^{\prime }}=l_{3}\varepsilon_{i3}\;\mathrm{and}\;i=1,2,3$$

Set II: Edge

1. Edge AD, B′C′, BC and A′D′

(21) $$\begin{array}{l} u_{i}^{E-AD}-u_{i}^{E-B^{\prime }C^{\prime }}=l_{1}\varepsilon_{i1}-l_{2}\varepsilon_{i2}\;\mathrm{and}\;i=1,2,3\\ u_{i}^{E-BC}-u_{i}^{E-A^{\prime }D^{\prime }}=l_{1}\varepsilon_{i1}+l_{2}\varepsilon_{i2}\;\mathrm{and}\;i=1,2,3 \end{array}$$

2. Edge AB, D′C′, DC and A′B′

(22) $$\begin{array}{l} u_{i}^{E-AB}-u_{i}^{E-D^{\prime }C^{\prime }}=l_{1}\varepsilon_{i1}+l_{3}\varepsilon_{i3}\;\mathrm{and}\;i=1,2,3\\ u_{i}^{E-DC}-u_{i}^{E-A^{\prime }B^{\prime }}=l_{1}\varepsilon_{i1}-l_{3}\varepsilon_{i3}\;\mathrm{and}\;i=1,2,3 \end{array}$$

3. Edge BB′, DD′, CC and AA′

(23) $$\begin{array}{l} u_{i}^{E-BB^{\prime }}-u_{i}^{E-DD^{\prime }}=l_{2}\varepsilon_{i2}+l_{3}\varepsilon_{i3}\;\mathrm{and}\;i=1,2,3\\ u_{i}^{E-CC^{\prime }}-u_{i}^{E-AA^{\prime }}=l_{2}\varepsilon_{i2}-l_{3}\varepsilon_{i3}\;\mathrm{and}\;i=1,2,3 \end{array}$$

Set III: Corner

1. Corner A, C′, A′, C, B, D′, B′ and D

(24) $$\begin{array}{l} u_{i}^{C-A}-u_{i}^{C-C^{\prime }}=l_{1}\varepsilon_{i1}-l_{2}\varepsilon_{i2}+l\varepsilon_{i3}\;\mathrm{and}\;i=1,2,3\\ u_{i}^{C-A^{\prime }}-u_{i}^{C-C}=-l_{1}\varepsilon_{i1}-l_{2}\varepsilon_{i2}+l_{3}\varepsilon_{i3}\;\mathrm{and}\;i=1,2,3\\ u_{i}^{C-B}-u_{i}^{C-D^{\prime }}=l_{1}\varepsilon_{i1}+l_{2}\varepsilon_{i2}+l_{3}\varepsilon_{i3}\;\mathrm{and}\;i=1,2,3\\ u_{i}^{C-D}-u_{i}^{C-B^{\prime }}=l_{1}\varepsilon_{i1}-l_{2}\varepsilon_{i2}-l_{3}\varepsilon_{i3}\;\mathrm{and}\;i=1,2,3 \end{array}$$

Equations (18)–(24) represent the PBC applied to solve the equivalent mechanical properties of the rSVE using FEA. After a certain given strain $\varepsilon_{ij}^{0}$ is imposed on the boundary using Equations (18)–(24), a complex strain state will arise within the rSVE. However, according to Equation (2), the volumetric average of the strain within the rSVE is equal to the imposed given strain, that is:

(25) $${\bar{\varepsilon }}_{ij}=\frac{1}{V}\int_{\Omega }\varepsilon_{ij}dV=\varepsilon_{ij}^{0}$$

For the homogenized composite structure, the relationship between average stress and strain is given by:

(26) $${\bar{\sigma }}_{\alpha }=C_{\alpha \beta }{\bar{\varepsilon }}_{\beta }$$

where i,j=1,…,3 and α,β=1,…,6 utilize the Voigt notation. By solving the elasticity model with the boundary condition equations in the form of Equations (18)–(24), all components of the stiffness matrix ***C*** can be determined. To facilitate numerical implementation, by applying a unit strain $\varepsilon_{\beta }^{0}=1$, the stress field $\sigma_{\alpha }$ can be solved, and its average value corresponds to the required components of the stiffness matrix. Imposing a unit strain once allows for the calculation of one column of the stiffness matrix components, that is:

(27) $$C_{\alpha \beta }={\bar{\sigma }}_{\alpha }=\frac{1}{V}\int_{\Omega }\sigma_{\alpha }dV\;\mathrm{with}\;\varepsilon_{\beta }^{0}=1$$

Within the FEA, the Gauss-Legendre quadrature is implemented for numerical integration across each finite element. To facilitate comprehension, Table 1 delineates the procedural guidelines for imposing the boundary conditions as dictated by Equations (18)–(24) during the computation of the stiffness matrix ***C***. It is important to reiterate that these Equations (18)–(24) are founded upon a displacement-based formulation, which ensures that the stress boundary conditions are inherently fulfilled, thereby eliminating the necessity for their explicit imposition.

#### 3.2.2. Equivalent Coefficient of Thermal Expansion Evaluation

When determining the CTE of the rSVE, the calculation is grounded on the premise that a uniform thermal strain will not induce macroscopic stress within the rSVE when employing finite element homogenization methods to calculate the CTE of the multi-scale structure. A small uniform temperature increase with ΔT set to 1 is applied across the entire rSVE, and the reference temperature is considered to be at zero strain. Following the rules outlined in Table 2 and utilizing PBC, the equivalent CTE is calculated using the following formula:

(28) $$\alpha_{\alpha }=-\frac{C^{-1}{{\bar{\sigma }}^{\prime }}_{\alpha }}{\Delta T}$$

where ***C*** refers to the stiffness matrix that was calculated using the PBC as previously described. The variable ${{\bar{\sigma }}^{\prime }}_{\alpha }$ is determined by Equation (29) under the current BC:

(29) $${{\bar{\sigma }}^{\prime }}_{\alpha }=\frac{1}{V}\int_{\Omega }{\sigma^{\prime }}_{\alpha }dV\;\mathrm{with}\;\varepsilon_{\beta }^{0}=0$$

Similar to the computation of the stiffness tensor, Equation (13) can be expressed as:

(30) $$\begin{array}{l} T^{k+}=G_{i}^{0}x_{i}^{k+}+t\;\mathrm{on}\;\partial \Omega^{+}\\ \mathrm{and}\\ T^{k-}=G_{i}^{0}x_{i}^{k-}+t\;\mathrm{on}\;\partial \Omega^{-} \end{array}$$

Due to the periodicity, the difference in the aforementioned equation can be expressed as:

(31) $$T^{k+}-T^{k-}=G_{i}^{0}\left(x_{i}^{k+}-x_{i}^{k-}\right)=G_{i}^{0}\Delta x_{i}^{k}=\Delta T_{i}$$

The application of this equation ensures the continuity of the temperature field. Additionally, this equation represents a special type of thermal BC that specifies the temperature difference between two opposing boundaries, rather than prescribing a fixed temperature value at the boundaries.

#### 3.2.3. Equivalent Thermal Conductivity Evaluation

To assess the overall equivalent thermal conductivity tensor of the composite structure, the following BCs are applied by enforcement:

Set I: Surface

1. Surface ABCD and A′B′C′D′

(32) $$T^{S-ABCD}-T^{S-A^{\prime }B^{\prime }C^{\prime }D^{\prime }}=l_{1}\nabla T_{1}$$

2. Surface BCC′B′ and ADD′A′

(33) $$T^{S-BCC^{\prime }B^{\prime }}-T^{S-ADD^{\prime }A^{\prime }}=l_{2}\nabla T_{2}$$

3. Surface ABB′A′ and DCC′D′

(34) $$T^{S-ABB^{\prime }A^{\prime }}-T^{S-DCC^{\prime }D^{\prime }}=l_{3}\nabla T_{3}$$

Set II: Edge

1. Edge AD, B′C′, BC and A′D′

(35) $$\begin{array}{l} T^{E-AD}-T^{E-B^{\prime }C^{\prime }}=l_{1}\nabla T_{1}-l_{2}\nabla T_{2}\\ T^{E-BC}-T^{E-A^{\prime }D^{\prime }}=l_{1}\nabla T_{1}+l_{2}\nabla T_{2} \end{array}$$

2. Edge AB, D′C′, DC and A′B′

(36) $$\begin{array}{l} T^{E-AB}-T^{E-D^{\prime }C^{\prime }}=l_{1}\nabla T_{1}+l_{3}\nabla T_{3}\\ T^{E-DC}-T^{E-A^{\prime }B^{\prime }}=l_{1}\nabla T_{1}-l_{3}\nabla T_{3} \end{array}$$

3. Edge BB′, DD′, CC and AA′

(37) $$\begin{array}{l} T^{E-BB^{\prime }}-T^{E-DD^{\prime }}=l_{2}\nabla T_{2}+l_{3}\nabla T_{3}\\ T^{E-CC^{\prime }}-T^{E-AA^{\prime }}=l_{2}\nabla T_{2}-l_{3}\nabla T_{3} \end{array}$$

Set III: Corner

1. Corner A, C′, A′, C, B, D′, B′ and D

(38) $$\begin{array}{l} T^{C-A}-T^{C-C^{\prime }}=l_{1}\nabla T_{1}-l_{2}\nabla T_{2}+l_{3}\nabla T_{3}\\ T^{C-A^{\prime }}-T^{C-C}=-l_{1}\nabla T_{1}-l_{2}\nabla T_{2}+l_{3}\nabla T_{3}\\ T^{C-B}-T^{C-D^{\prime }}=l_{1}\nabla T_{1}+l_{2}\nabla T_{2}+l_{3}\nabla T_{3}\\ T^{C-D}-T^{C-B^{\prime }}=l_{1}\nabla T_{1}-l_{2}\nabla T_{2}-l_{3}\nabla T_{3} \end{array}$$

For materials with anisotropic thermal conductivity, Fourier’s law establishes the following relationship between the heat flux and the temperature gradient:

(39) $${\bar{q}}_{\alpha }=-k_{\alpha \beta }\nabla T_{\beta }$$

where $k_{\alpha \beta }$ represents the thermal conductivity tensor. To obtain the equivalent thermal conductivity tensor of the rSVE, the above equation can be written as:

(40) $$k_{\alpha \beta }=-\frac{{\bar{q}}_{\alpha }}{\nabla T_{\beta }}$$

When a given temperature gradient $\nabla T^{0}$ is applied on the boundaries using Equations (32)–(38), a complex heat flux will arise within the rSVE. For ease of numerical implementation, a unit temperature gradient $\nabla T^{0}=1$ is typically prescribed, allowing for the calculation of the average heat flux across the entire rSVE. Applying a unit temperature gradient once yields a column of the thermal conductivity tensor, that is:

(41) $$k_{\alpha \beta }=-{\bar{q}}_{\alpha }=-\frac{1}{V}\int_{\Omega }q_{\alpha }dV\;\mathrm{with}\;\nabla T_{\beta }^{0}=1$$

Similarly, within each element, the calculation is performed using the Gauss-Legendre integration method.

Table 3 provides the application rules for the BCs in Equations (32)–(38) when calculating the equivalent thermal conductivity tensor of the rSVE.

### 3.3. Validation of the Numerical Homogenization Method

#### 3.3.1. Verification of Continuity Conditions of the PBC

**Figure 6:**
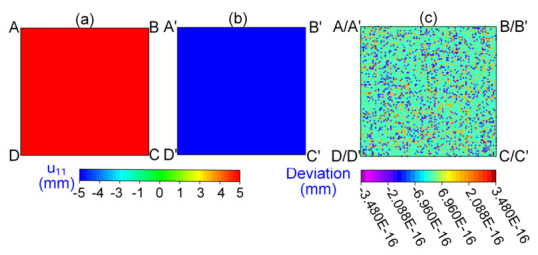
Distance U_11_ of the corresponding nodes located on two opposite surfaces S-ABCD and S-A′B′C′D′ and their relative deviations: (**a**) Distance U_11_ on the S-ABCD; (**b**) Distance U_11_ on the S-A′B′C′D′; (**c**) relative deviation of Distance U_11_.

To verify the correctness of the PBC, it is necessary to examine the continuity of displacement, stress, strain, and thermal flux on the boundaries of the rSVE. The rSVE model of the RDL is chosen for verification due to its geometric intricacy, offering a stringent test case for general cases. Initially, periodic congruence of the BC (i.e., the continuity of displacement) is checked. For brevity, the study focuses on the displacement U_11_ at corresponding nodes on the collinear opposing surfaces S-ABCD and S-A′B′C′D′ under tensile loading in the z-direction. The displacements U_11_ at the corresponding nodes on surfaces S-ABCD and S-A′B′C′D′, generated by the prescribed tensile load in the z-direction, are shown in Figure 6. Upon examining the displacement contour depicted in Figure 6, it is observed that after the application of the PBC, the magnitudes of displacement on parallel and opposite surface pairs are consistent. The relative displacement deviation in Figure 6c also indicates that the displacement difference between these two surfaces is to the extent that it can be considered zero. Furthermore, it is confirmed that the displacement U_11_ at corresponding nodes on these two opposite surface pairs is constant, which satisfies Equation (16). This confirms the continuity of displacement at corresponding nodes on the two opposite surface pairs of the rSVE provided by the applied PBC.

**Figure 7:**
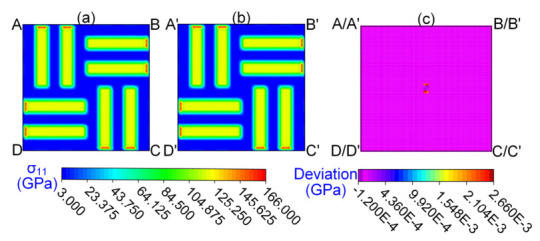
Stress *σ*_11_ of the corresponding nodes located on two opposite surfaces S-ABCD and S-A′B′C′D′ and their relative deviations: (**a**) Stress *σ*_11_ on the S-ABCD; (**b**) Stress *σ*_11_ on the S-A′B′C′D′; (**c**) relative deviation of stress *σ*_11_.

**Figure 8:**
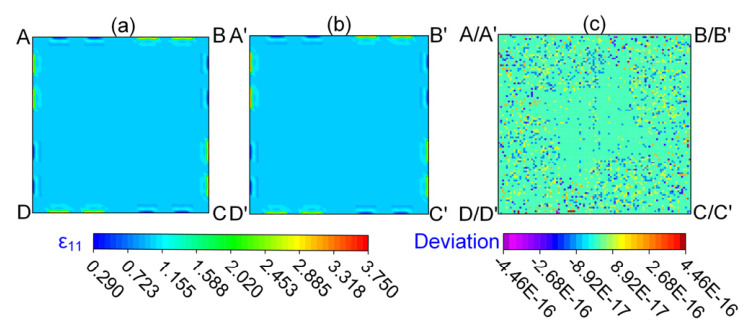
Strain *ε*_11_ of the corresponding nodes located on two opposite surfaces S-ABCD and S-A′B′C′D′ and their relative deviations: (**a**) strain *ε*_11_ on the S-ABCD; (**b**) strain *ε*_11_ on the S-A′B′C′D′; (**c**) relative deviation of strain *ε*_11_.

Figure 7 and Figure 8 scrutinize the continuity conditions of stress and strain at the corresponding nodes on the rSVE boundary. Similarly, for the sake of brevity, only the continuity conditions of stress $\sigma_{11}$ and strain $\varepsilon_{11}$ at corresponding nodes on the opposite surface pairs S-ABCD and S-A′B′C′D′ under tensile loading in the z-direction are illustrated. The stress $\sigma_{11}$ and strain $\varepsilon_{11}$ are shown in Figure 7 and Figure 8, which also include the relative deviations of these stresses and strains at the corresponding nodes. Please note that the displacement, stress, and strain values at the nodes are evaluated from the corresponding Gaussian integration points using the shape functions of the elements concurrent at the nodes. The results in Figure 7c and Figure 8c demonstrate consistent trends, with the results on surfaces S-ABCD and S-A′B′C′D′ being nearly identical for both stress and strain, and the deviations being sufficiently small enough to be considered negligible.

**Figure 9:**
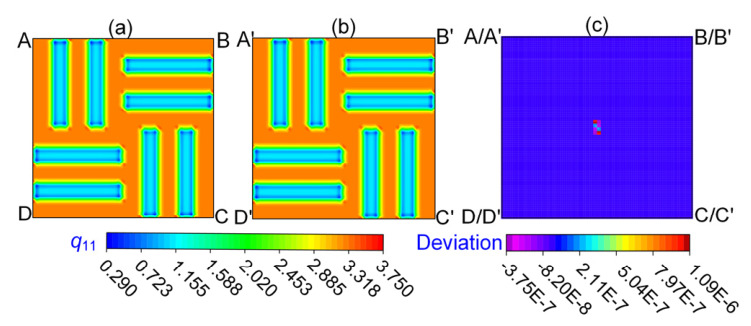
Thermal flux *q*_11_ of the corresponding nodes located on two opposite surfaces S-ABCD and S-A′B′C′D′ and their relative deviations: (**a**) thermal flux *q*_11_ on the S-ABCD; (**b**) thermal flux *q*_11_ on the S-A′B′C′D′; (**c**) relative deviation of thermal flux *q*_11_.

Figure 9 scrutinizes the continuity conditions of the heat flux at the boundary nodes on collinear opposing surfaces of the rSVE. As evidenced in the figure, the heat flux at the corresponding node on collinear surfaces S-ABCD and S-A′B′C′D′ is almost the same, and the deviation is negligible, which indicates that the proposed periodic boundary application method meets the continuity of heat flux.

While the continuity verification is exemplified here for a representative normal loading case (z-direction tension) on the most complex RDL geometry, it should be noted that the PBC constraints are linear and predicated solely on geometry. The rigorous extraction of the symmetric and positive definite stiffness matrix ***C***_eff_ (in MPa) shown in Equation (42) requires the application of both normal and shear macroscopic strains, which explicitly verifies the correctness of the PBC under all loading conditions. Consequently, the single-case demonstration is mathematically rigorous to confirm the robustness of the numerical implementation. Furthermore, note that the off-diagonal entries in the 4th–6th rows and columns of ***C***_eff_ are more than nine orders of magnitude smaller than the other entries, and are thus numerically null.

(42) $$C_{\mathrm{eff}}=\left[\begin{array}{llllll} 5.069\times 10^{4} & 3.952\times 10^{3} & 3.952\times 10^{3} & 3.600\times 10^{-9} & -6.447\times 10^{-10} & 1.764\times 10^{-10}\\ 3.952\times 10^{3} & 9.494\times 10^{3} & 2.132\times 10^{3} & 4.962\times 10^{-6} & -1.170\times 10^{-9} & -2.308\times 10^{-10}\\ 3.952\times 10^{3} & 2.132\times 10^{3} & 9.494\times 10^{3} & -4.964\times 10^{-6} & -2.226\times 10^{-10} & 3.984\times 10^{-10}\\ -2.980\times 10^{-11} & 5.159\times 10^{-8} & -5.172\times 10^{-8} & 1.612\times 10^{3} & 3.483\times 10^{-12} & 5.913\times 10^{-11}\\ -5.271\times 10^{-11} & -1.508\times 10^{-10} & 6.631\times 10^{-10} & -8.257\times 10^{-10} & 2.819\times 10^{3} & 4.458\times 10^{-7}\\ 9.145\times 10^{-10} & -7.570\times 10^{-10} & 5.440\times 10^{-10} & -2.924\times 10^{-10} & -4.453\times 10^{-7} & 2.819\times 10^{3} \end{array}\right]$$

#### 3.3.2. Accuracy Verification of PBC-Based Numerical Homogenization

To further corroborate the accuracy of the PBC-based numerical homogenization strategy, this validation exercise benchmarks it against previously reported results for effective elastic modulus, effective CTE, and effective thermal conductivity.

**Figure 10:**
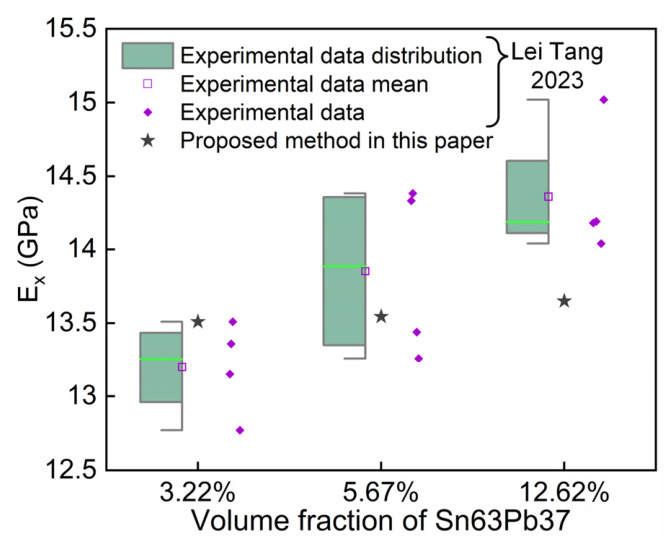
Comparison of the equivalent transverse elastic modulus obtained by the method in this paper and the method in [20].

The predictive fidelity for the transverse elastic modulus was first validated using a periodic composite structure consisting of PCB, micro bumps, and underfill [20]. The PCB, made of FR-4, had a thickness of 1 mm; the micro bumps, made of Sn63Pb37, had a height of 0.34 mm; and the underfill was an adhesive. The elastic moduli of the PCB, Sn63Pb37, and underfill were 14.48 GPa, 25.8 GPa, and 7.3 GPa, respectively. The benchmark specimen comprised two PCB layers with micro bumps encapsulated by the underfill. By adjusting the in-plane dimensions of the rSVE, three micro bump volume fractions were obtained: 3.22%, 5.67%, and 12.62%. The equivalent transverse elastic modulus *E_x_* was then computed using the PBC-based numerical homogenization method proposed in this study and compared with existing experimental data [20]. As shown in Figure 10, for the three volume fractions, the deviations between the calculated values and the experimental means were 2.27%, −2.21%, and −4.94%, respectively, indicating excellent concordance. These results confirm the accuracy of the proposed method for modulus homogenization.

**Figure 11:**
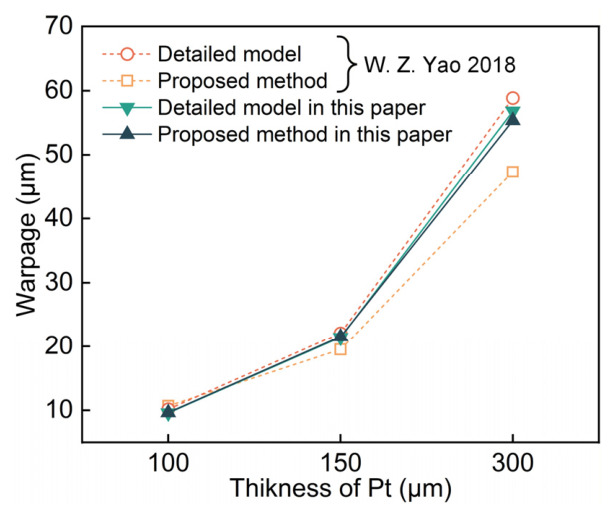
Comparison of the warpage obtained by the method in this paper and the method in [21].

For the CTE validation, a multilayer structural model from the literature [21] was employed. This model consists of a 500 µm thick silicon substrate upon which two uniform thin films—undoped silica glass (USG) and Pt—are deposited. The USG film has a thickness of 400 nm, while the Pt film thickness is varied at 100 nm, 150 nm, and 300 nm. A thermal load corresponding to the final stage of a full thermal cycle (cooling from 450 °C to 22 °C) was applied. Since the thicknesses of the USG and Pt films are far smaller than that of the silicon substrate, an equivalent single-layer film was adopted for analytical efficiency, which necessitates the calculation of its equivalent CTE. The PBC-based numerical homogenization method proposed in this work was compared with the CTE equivalence approach reported in [21], using the distal-end warpage as the benchmark, as shown in Figure 11. It can be observed that the warpage difference between the homogenized equivalent model and the detailed model is negligible. For the 100 nm Pt layer, the warpage obtained from the homogenized model differs by 0.48% from that of the detailed model; for the 150 nm Pt layer, the difference is also approximately 0.48%; and for the 300 nm Pt layer, the difference is −2.58%. In contrast, the accuracy of the method in [21] deteriorates markedly as the Pt layer thickness increases, reaching a warpage difference of 19.63% for the 300 nm Pt layer case. These results demonstrate that the proposed method can faithfully capture the equivalent CTE.

**Figure 12:**
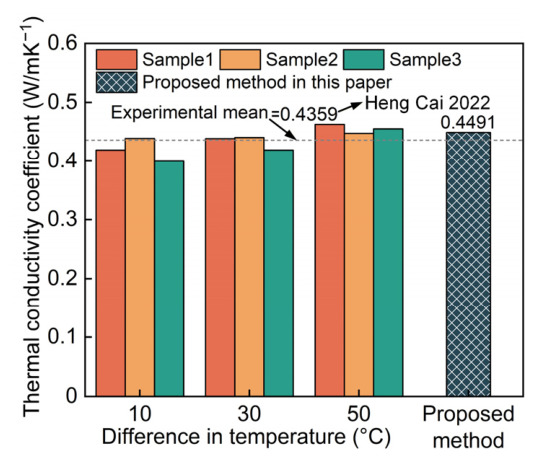
Comparison of the equivalent thermal conductivity coefficient obtained by the method in this paper and the method in [22].

For the validation of the effective thermal conductivity, a glass fiber-reinforced phenolic epoxy resin composite was employed [22]. The thermal conductivities of the glass fiber and the phenolic epoxy resin are 1.09 W/mK^−1^ and 0.20 W/mK^−1^, respectively. The circular fibers are positioned at the geometric center of the RVE, and a fiber volume fraction of V*_f_* = 0.55 was utilized. Using the PBC-based numerical homogenization method proposed in this work, the equivalent thermal conductivity of the composite was calculated and compared with the experimental measurements reported in [22]. As shown in Figure 12, the experimental mean thermal conductivity is 0.4359 W/mK^−1^, whereas the equivalent value computed by the proposed method is 0.4491 W/mK^−1^, giving a difference of only 3.03%. This congruence confirms the accuracy of the proposed method for thermal conductivity homogenization.

It should be acknowledged that the three validation cases presented above are sourced from material systems that differ from the specific 2.5D package structures (TSV, RDL, microbump layers) analyzed in Section 4. These cases serve to verify the mathematical rigor and numerical implementation of the PBC-based homogenization framework, rather than to replicate the exact geometries of TSVs or RDLs. Since the PBC formulations are agnostic to geometry, successful validation against experimental data for these three distinct composite systems provides confidence that the method is correctly implemented and can be reliably applied to other periodic microstructures, including those in 2.5D packages. Direct experimental validation on full 2.5D package structures is slated for subsequent investigation.

## 4. Application of PBC-Based Homogenization Method in 2.5D Packaging Manufacturing and Service

**Figure 13:**
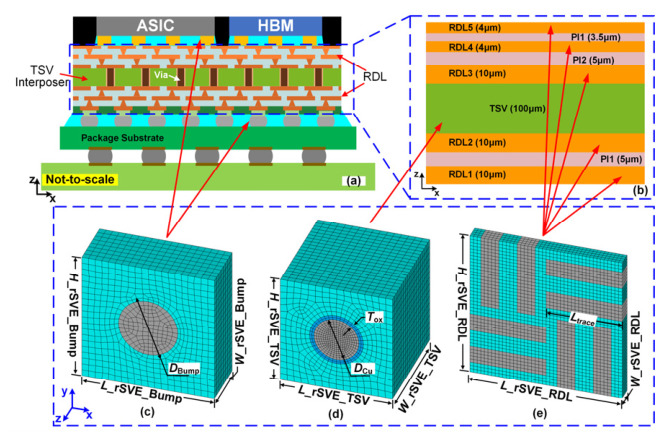
2.5D package structure and its rSVEs: (**a**) multi-scale structure in 2.5D package; (**b**) simplified cross-section diagram of TSV interposer with RDLs; (**c**) the rSVE of microbump; (**d**) the rSVE of TSV; (**e**) the rSVE of RDL.

The multi-scale structure in the 2.5D package presents challenges that undermine the efficacy of traditional analysis methods in an effective way. In contrast, the PBC-based numerical homogenization method can significantly improve computational throughput. This section showcases the application of this method to both the manufacturing process and the operating conditions of the 2.5D package. Figure 13 illustrates the rSVE models representing the complex multi-scale structure. Figure 13a shows a schematic depiction of the overall 2.5D package, Figure 13b provides a representative layout of the TSV interposer with multiple RDLs, and Figure 13c presents the rSVE models for the microbump layer and the C4 bump layer. These two layers share a similar structure, consisting of an underfill matrix with microbumps or C4 bumps distributed within. Figure 13d displays the rSVE model of the TSV layer, where the silicon substrate is embedded with Cu conductors and SiO_2_ insulating layers. For the RDL layer, which features densely packed, fine-pitch traces, a credible equivalent structure adopted from the literature [23,24] serves as the rSVE, in which Cu traces are arranged in an orthogonal pattern within a photosensitive polyimide matrix.

### 4.1. Warpage Analysis for Wafer-Level Manufacturing Process

The fabrication of the TSV interposer constitutes a pivotal phase in the wafer-level packaging process. The TSV interposer is constructed by stacking a TSV layer, multiple RDLs, and photosensitive PI dielectric layers, resulting in an intricate architecture. The configuration of copper conductors and the I/O count within the TSV interposer dictate the opening positions in the PI and the routing of the RDL traces. Constructing a detailed finite element model for even a single RDL layer is prohibitively arduous, primarily owing to the multi-scale nature of the geometric features involved. It is therefore imperative to employ the PBC-based numerical homogenization method to represent the mechanical properties of these complex multi-scale wafer-level package structures as equivalent properties. This approach facilitates thermomechanical numerical simulations and expedites numerical convergence.

This study initially examines the warpage of the TSV interposer during wafer-level packaging induced by the process thermal profile. The TSV interposer has in-plane dimensions of 25 mm × 18 mm and incorporates vias with a diameter of 10 µm. Multilayer RDLs are implemented on both the front and back sides to route the vias to the bond pads designated for the top chip components and the bottom substrate. These TSV interposers are fabricated on a 12-inch (300 mm) wafer.

**Figure 14:**
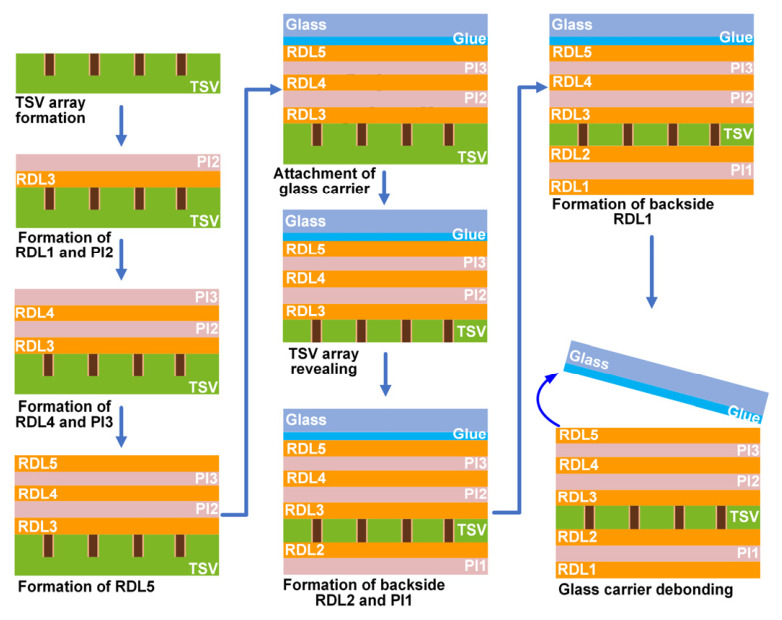
Fabrication process of TSV interposer with RDLs.

As illustrated in Figure 14, the first manufacturing step involves forming vias in the wafer and heating it to 200 °C for Cu electroplating. The second step entails the fabrication of RDL3 and the second PI dielectric layer (PI2), followed by curing at 230 °C. The third step involves the fabrication of RDL4 and the third PI dielectric layer (PI3), also cured at 230 °C, but with finer wiring. RDL5, which provides the bridge to the application-specific integrated circuit (ASIC) and high-bandwidth memory (HBM), exhibits comparable miniaturization and is cured at the same temperature of 230 °C. Subsequently, a glass carrier is bonded to the front side using a bonding adhesive. After carrier attachment, a grinding process is performed on the wafer backside to expose the TSVs. The conditions for fabricating the backside RDL2 and RDL1 are identical to those for RDL3 and are achieved by repeating the aforementioned steps twice. Process steps that have a negligible impact on warpage—such as seed layer deposition, photoresist patterning, photoresist stripping, and seed layer etching—are omitted from the simulation. The warpage evolution of the wafer during the interposer manufacturing process is modeled using a sequential element activation and deactivation scheme.

During the analysis, the PBC-based numerical homogenization method proposed in this study is leveraged to homogenize the TSV layer and the RDLs. The rSVE model for the TSV layer is shown in Figure 13d and consists of the silicon substrate, the SiO_2_ insulating layer, and the copper conductor. The rSVE model for the RDL is presented in Figure 13e and consists of copper traces and PI, with the copper traces uniformly distributed within the rSVE according to the designed volume fraction. Table 4 lists the material properties used in the analysis. In the subsequent homogenization procedure, these properties are assigned to the rSVEs. Table 5 gives the rSVE dimensions and metal volume fractions for the different structures. Subsequently, a mesh convergence study was performed for the rSVE of RDL1, and the results are shown in Table 6. While preserving four significant figures and balancing computational efficiency with accuracy, the mesh size for RDL1 was determined to be 2 μm. On this basis, the rSVE mesh sizes for the TSV layer and RDL4 were set to 1 μm and 0.5 μm, respectively. It should be noted that in the wafer-level warpage simulation, the temperature-dependent CTE of PI (Table 4) is fully accounted for. The effective CTE of each rSVE is computed at each relevant temperature point using the PBC-based homogenization method. Table 7 reports the equivalent CTE values at 25 °C, 50 °C, 100 °C, 150 °C, 200 °C, and 250 °C for all rSVE components. The temperature-dependent effective CTEs are then assigned to the corresponding homogenized layers in the process simulation. This ensures that the thermal expansion behavior of the RDLs is accurately captured across the entire fabrication temperature range.

It should be noted that in the simulation, the reference temperature (i.e., the stress-free state) for each material is taken as its corresponding process temperature. The RDL, however, presents a special case: the Cu traces are assumed to be electroplated at 25 °C, whereas the PI is cured at 230 °C. It is therefore necessary to determine an equivalent stress-free temperature for the RDL. To this end, a zero-strain-based method is adopted [23,24]. The fundamental idea of this method is to assume that, in the case of Cu and PI materials connected in series, when the equivalent reference temperature *T*_ref_eq_ is reached, the overall deformation and strain caused by the expansion or contraction of Cu and PI materials along their in-plane orientation should be zero. Subsequently, *T*_ref_eq_ can be solved and mathematically expressed as [23,24]:

(43) $$T_{\mathrm{ref}\text{\_}\mathrm{eq}}=\frac{V_{\mathrm{Cu}}\alpha_{\mathrm{Cu}}T_{\mathrm{ref}\text{\_}\mathrm{Cu}}+V_{\mathrm{PI}}\alpha_{\mathrm{PI}}T_{\mathrm{ref}\text{\_}\mathrm{PI}}}{V_{\mathrm{Cu}}\alpha_{\mathrm{Cu}}+V_{\mathrm{PI}}\alpha_{\mathrm{PI}}}$$

where $V_{\mathrm{Cu}}$ and $V_{\mathrm{PI}}$ represent the volume fractions of Cu and PI in RDL, respectively, with $V_{\mathrm{PI}}=1-V_{\mathrm{Cu}}$; $\alpha_{\mathrm{Cu}}$ and $\alpha_{\mathrm{PI}}$ represent the thermal expansion coefficients of Cu and PI, respectively; $T_{\mathrm{ref}\text{\_}\mathrm{Cu}}$ and $T_{\mathrm{ref}\text{\_}\mathrm{PI}}$ represent the process reference temperatures of Cu and PI, respectively.

**Figure 15:**
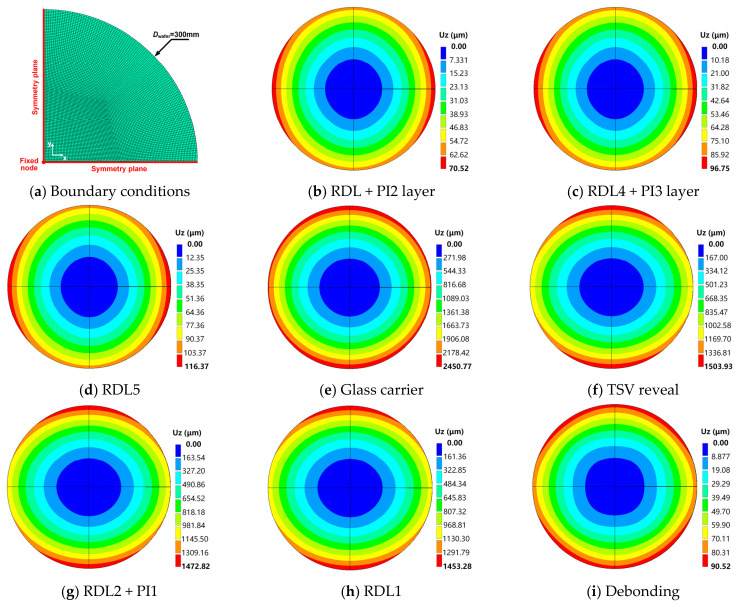
Finite element model for process-induced warpage analysis and warpage contours of wafer-level fabrication. (**a**) Finite element model for process-induced warpage analysis; (**b**–**i**) the warpage contour of the wafer after each process phase.

The homogenized finite element model of the wafer is shown in Figure 15a. The simulation follows the manufacturing sequence using the equivalent model. By exploiting symmetry, the computation time is reduced by modeling only one quarter of the wafer. Accordingly, symmetry boundary conditions are imposed on the yz- and xz-planes, and the wafer center is constrained in the z-direction to prevent rigid-body motion. Figure 15b–i displays the warpage contours of the wafer after each process step.

#### 4.1.1. Effect of Backside RDL on Warpage

**Figure 16:**
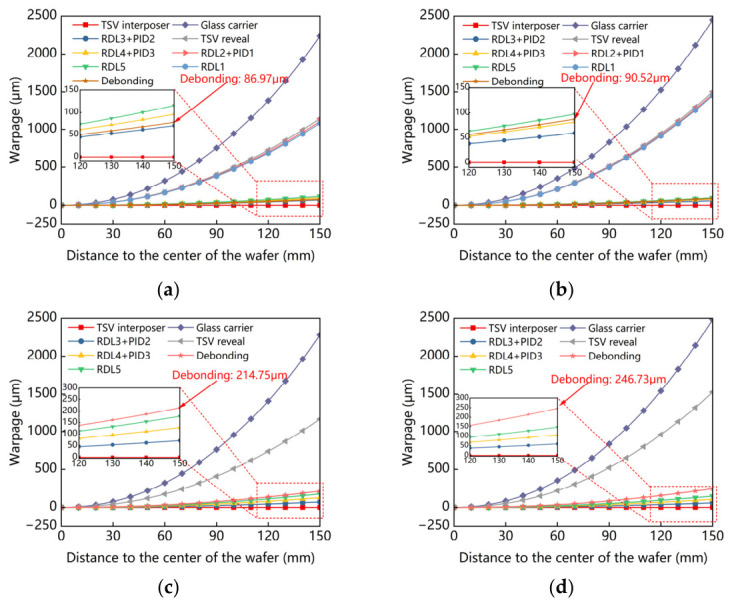
Effect of different processes on wafer warpage. (**a**) Wafer warpage in the x-direction with backside RDL; (**b**) wafer warpage in the y-direction with backside RDL; (**c**) wafer warpage in the x-direction without backside RDL; (**d**) wafer warpage in the y-direction without backside RDL.

The influence of the backside RDL on wafer warpage was first assessed. The backside RDL consists of RDL1 and RDL2, and its design intent is to redistribute the I/Os to designated landing sites for subsequent interconnection. As shown in Figure 16, the out-of-plane (z-direction) warpage of the wafer was extracted along the x- and y-axes after each process step. With the backside RDL present, the maximum warpage after wafer debonding reaches 86.97 µm in the x-direction and 90.52 µm in the y-direction. In contrast, when the backside RDL is absent, the pronounced thermo-mechanical mismatch among the TSV layer, RDLs, and PI layers results in far more severe warpage; the corresponding maximum values after debonding are 214.75 µm in the x-direction and 246.73 µm in the y-direction. These results indicate that the CTE mismatch between the different material layers drives wafer warpage, and that the backside RDL mitigates this stress mismatch. The interposer configuration with the backside RDL exhibits a geometrically symmetric laminate structure, which helps reduce warpage during fabrication. Additionally, as can be seen from the warpage contours in Figure 16, the largest warpage throughout the entire process occurs after the temporary bonding of the glass carrier. This is attributed to the substantial CTE incompatibility between the CTE of the glass carrier and those of the other materials. Although temporary glass carrier bonding is an intermediate process step, the excessive warpage it introduces may cause interposer die shift and lead to mechanical reliability concerns.

#### 4.1.2. Effect of Cu Volume Fraction in TSV Layer on Warpage

**Figure 17:**
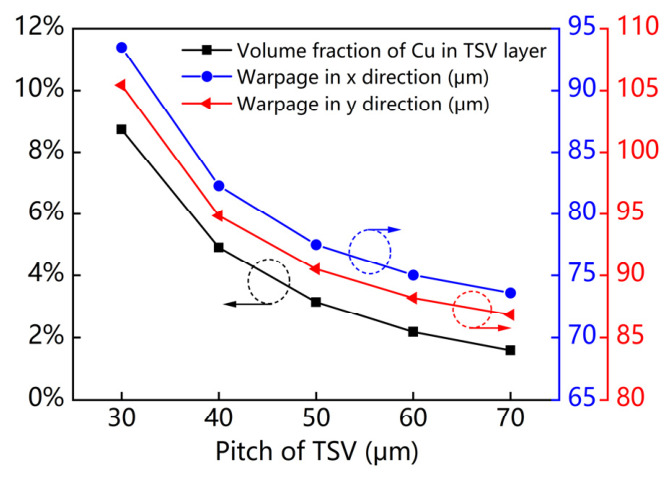
Impact of Copper Volume Fraction in TSV Layer on Warpage Behavior.

This study quantifies the influence of the Cu volume fraction in the TSV layer on wafer warpage by systematically varying the TSV pitch from 30 µm to 70 µm. As the TSV pitch increases, the Cu volume fraction decreases. The corresponding equivalent mechanical properties of the TSV layer are listed in Table 8. The results show that both the in-plane and out-of-plane elastic moduli exhibit a monotonic increase, whereas the CTE declines progressively. Regarding the maximum wafer warpage after debonding, as shown in Figure 17, increasing the TSV pitch attenuates the maximum warpage in the x-direction from 93.50 µm to 73.59 µm and in the y-direction from 105.43 µm to 86.82 µm.

#### 4.1.3. Effect of SiO_2_ Thickness in TSV Layer on Warpage

**Figure 18:**
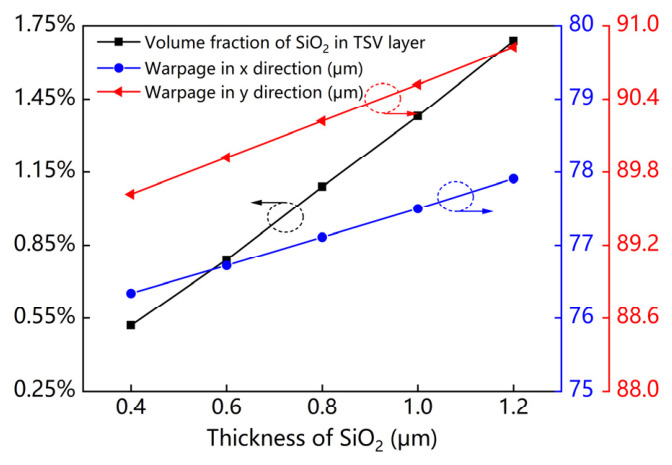
Influence of SiO_2_ Layer Thickness on Warpage in TSV Structures.

Table 9 summarizes the variation in the equivalent mechanical properties of the TSV layer with increasing SiO_2_ thickness. Because the elastic modulus of SiO_2_ is orders of magnitude lower than that of the silicon substrate, an increase in the SiO_2_ thickness from 0.4 µm to 1.2 µm elevates the SiO_2_ volume fraction, which in turn diminishes both the in-plane and out-of-plane elastic moduli as well as the CTE. As a consequence, the maximum wafer warpage after debonding manifests a marginal elevation, as shown in Figure 18: from 76.34 µm to 77.91 µm in the x-direction and from 89.62 µm to 90.83 µm in the y-direction.

#### 4.1.4. Effect of TSV Array Layout on the Homogenization of Physical Properties

**Figure 19:**
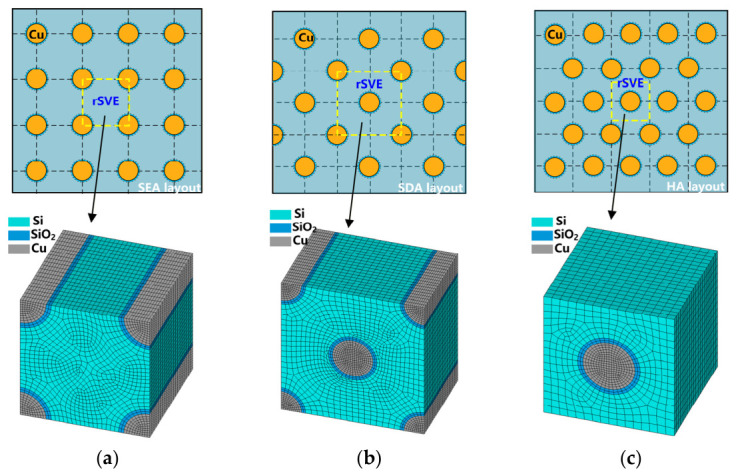
Different TSV array layouts and their rSVE configurations. (**a**) SEA layout; (**b**) SDA layout; (**c**) HA layout.

In engineering practice, three canonical array layouts are typically employed for TSV interposers: the square edge array (SEA), the staggered diagonal array (SDA), and the hexagonal array (HA). In the SEA layout, TSVs are uniformly arranged in a square grid with aligned edges; this layout is geometrically straightforward, which facilitates fabrication and lithographic alignment. The SDA layout places TSVs in a staggered pattern along both horizontal and vertical directions, forming a diagonally distributed grid. This configuration mitigates localized electromagnetic coupling between adjacent TSVs and improves signal integrity. The HA layout arranges TSVs in a hexagonal grid, promoting exceptional density uniformity, high geometric space utilization, and strong symmetry; however, it imposes heightened process complexity. The corresponding rSVE partitions for these three layouts are illustrated in Figure 19. To further assess the applicability of the proposed PBC-based method, the influence of different TSV array layouts and rSVE definitions on the equivalent physical properties is investigated.

**Figure 20:**
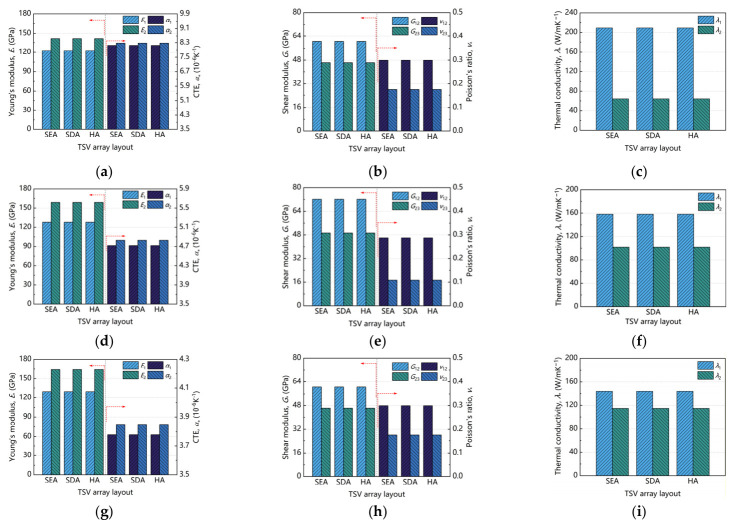
Effective physical parameters of TSV under different array layouts. (**a**) SEA layout with *V*_Cu_ = 8.73%; (**b**) SDA layout with *V*_Cu_ = 8.73%; (**c**) HA layout with *V*_Cu_ = 8.73%; (**d**) SEA layout with *V*_Cu_ = 3.14%; (**e**) SDA layout with *V*_Cu_ = 3.14%; (**f**) HA layout with *V*_Cu_ = 3.14%; (**g**) SEA layout with *V*_Cu_ = 1.60%; (**h**) SDA layout with *V*_Cu_ = 1.60%; (**i**) HA layout with *V*_Cu_ = 1.60%.

To demonstrate the generality of the proposed method, three cases with distinct material titrations were selected across the three array layouts: (1) 8.73% Cu, 3.84% SiO_2_, and 87.43% Si; (2) 3.14% Cu, 1.38% SiO_2_, and 95.48% Si; and (3) 1.60% Cu, 0.71% SiO_2_, and 97.69% Si (all by volume). Figure 20 compares the equivalent properties of the different array layouts. It can be observed that, given identical constituent volume fractions, the equivalent mechanical properties, CTE, and thermal conductivity are virtually indistinguishable across the three layouts. The marginal discrepancies arise from residual discretization artifacts among the rSVE models, which introduce numerically insignificant errors through nodal interpolation and extrapolation. This indicates that, provided the volume fractions of all constituents in the rSVE match those of the macroscopic model, the specific morphology of the rSVE exerts no discernible influence on the computed equivalent physical properties. Since the equivalent properties are effectively decoupled from the specific TSV array topology—as long as constituent volume fractions are conserved—a computationally expedient canonical square rSVE may be adopted for homogenization irrespective of the actual array type. In this study, we advocate utilizing the simple square unit cell illustrated in Figure 19c (employed here in the HA case). This rSVE geometry is topologically regular, readily meshable, and amenable to periodic boundary condition implementation, thereby further enhancing computational throughput. This recommendation pertains strictly to the rSVE geometry for homogenization and remains valid irrespective of the underlying complexity of the TSV array layout.

#### 4.1.5. Convergence and Efficiency Assessment of the Homogenized Wafer-Level FEA

To ensure the reliability of the macroscopic warpage predictions presented in the following parametric studies, a mesh sensitivity study was performed for the continuum-level wafer-level finite element model (quarter-symmetry model of the 300 mm wafer, as shown in Figure 15a). Four mesh densities were systematically evaluated, and the maximum out-of-plane warpage after debonding was used as the convergence criterion. As summarized in Table 10, the coarse mesh (~45,000 elements) already captures the general magnitude of warpage but deviates by approximately 5% from the very fine mesh solution. The medium mesh (~180,000 elements) limits the discrepancy to less than 0.6% compared with the very fine mesh (~1,440,000 elements), while the fine mesh (~720,000 elements) yields results statistically congruent with the baseline (deviation < 0.05%). Balancing numerical fidelity against computational expenditure, the medium mesh was adopted for all subsequent wafer-level simulations reported in Section 4.1.1, Section 4.1.2, Section 4.1.3 and Section 4.1.4.

It is worth noting that even the coarse mesh correctly captures the qualitative deformation characteristics, further demonstrating the robustness of the homogenization approach. Beyond accuracy, the homogenized model offers orders-of-magnitude enhancement in computational efficiency compared with a fully resolved heterogeneous counterpart. For the same 300 mm wafer geometry, a detailed model resolving all TSVs, RDL traces, and PI openings individually would require over 50 million elements and an estimated computation time exceeding 20 h with memory usage above 64 GB. In contrast, the present homogenized model with the medium mesh requires only 180,000 elements and completes the entire multi-step process simulation within approximately 45 min using 8 GB of memory. This corresponds to a reduction of more than 99% in element count and a speedup of over 25 times compared to the detailed model, confirming that the proposed PBC-based homogenization method not only delivers converged and accurate results but also provides a computationally tractable and industrially viable paradigm for full-scale 2.5D package process simulation.

In addition, it is acknowledged that the present simulation assumes temperature-invariant elastic moduli and CTE for materials other than PI and employs a linear elastic constitutive law for PI without explicitly accounting for viscoelastic stress relaxation. This simplification is rigorously justified based on the following reasons: (1) the elastic modulus of Cu varies by only 3–5% over the process temperature range, and Cu is present at relatively low volume fractions in the homogenized layers; (2) the CTE of PI—the material with the most significant temperature-dependent behavior—is fully accounted for in the warpage simulation; and (3) experimental studies have shown that PI cure shrinkage has little effect on warpage compared to CTE mismatch [25]. However, the primary objective of this work is to demonstrate the PBC-based homogenization methodology, and the warpage analysis serves as a benchmarking vehicle rather than a high-accuracy absolute prediction. All parametric comparisons are performed under consistent assumptions, so the relative trends—such as the effect of backside RDL, Cu volume fraction, and SiO_2_ thickness—remain physically robust. Incorporating full temperature-dependent properties and viscoelastic constitutive models for all materials is planned as future work to further improve absolute prediction accuracy.

### 4.2. Thermal Analysis for 2.5D Package Service Process

**Figure 21:**
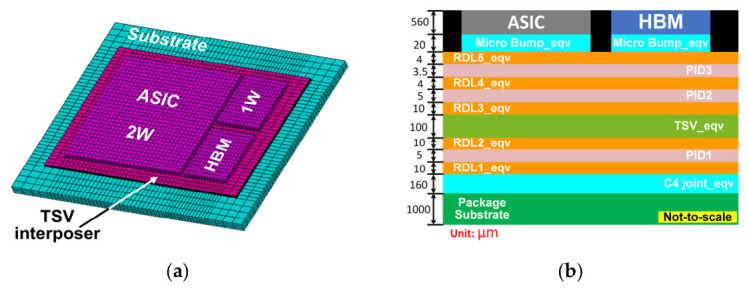
Schematics of 2.5D package for thermal analysis. (**a**) Thermal model of 2.5D package (removal of EMC and PCB for illustration); (**b**) homogenized structure of 2.5D package for thermal analysis.

In this section, the PBC-based numerical homogenization framework is deployed for the thermal analysis of the 2.5D package. The three-dimensional simulation model used for the thermal evaluation is shown in Figure 21. The method is employed to determine the in-plane and out-of-plane effective thermal conductivities of the microbump bonding layers, C4 bonding layers, underfill layers, and the TSV interposer featuring double-sided RDLs. Table 11 lists the thermal conductivities of the constituent materials in the 2.5D package. The geometric and topological parameters of the rSVEs used for extracting the equivalent thermal conductivity are provided in Table 12, and the homogenized effective thermal conductivities of each layer are presented in Table 13. The thermal analysis adopts the following boundary conditions: (i) the top surface of the ASIC and HBM chips is subjected to natural convection with a heat transfer coefficient of h = 10 W/(m^2^·K) and an ambient temperature of T_amb_ = 25 °C; (ii) the bottom surface of the PCB is constrained at T = 25 °C to represent board-level heat sinking; (iii) all other external surfaces are subjected to natural convection with h = 5 W/(m^2^·K) and T_amb_ = 25 °C; (iv) internal interfaces between layers are modeled as perfect bonded with negligible thermal contact resistance.

The heat generation rates are 2 W for the ASIC and 1 W for the HBM, uniformly distributed across their respective silicon volumes. Crucially, it is emphasized that these power levels correspond to the Thermal Design Power (TDP) specifications of the specific prototype chips used in this study. Specifically, the ASIC is a low-power controller ASIC designed for aerospace applications, and the HBM is a lower-capacity HBM2 module (4 GB) operating at reduced clock frequencies. These values are therefore representative of the specific package architecture under investigation and should not be interpreted as generic values for all HBM/ASIC packages.

**Figure 22:**
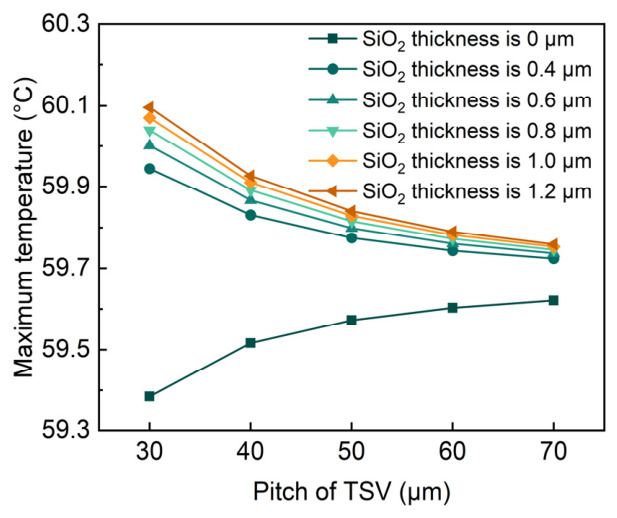
Effect of SiO_2_ thickness and pitch of TSV on the maximum chip temperature in 2.5D package.

**Figure 23:**
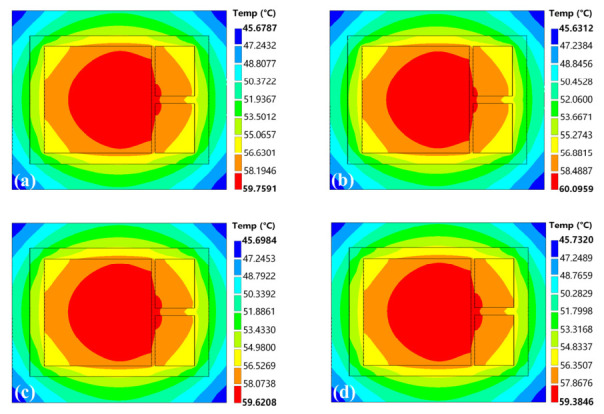
2.5D package temperature distribution contours (removal of EMC and PCB for illustration). (**a**) Pitch of TSV is 70 µm with 1.2 um SiO_2_ layer; (**b**) pitch of TSV is 30 µm with 1.2 um SiO_2_ layer; (**c**) pitch of TSV is 70 µm without SiO_2_ layer; (**d**) pitch of TSV is 30 µm without SiO_2_ layer.

Figure 22 shows the variation in the junction temperature of the 2.5D package as a function of TSV pitch and SiO_2_ insulating layer thickness, while Figure 23 presents the corresponding temperature contours. It is observed that the junction temperature increases with increasing SiO_2_ thickness, which is attributed to the intrinsically low thermal conductivity of SiO_2_. Counterintuitively, even when the volume fraction of the high-thermal-conductivity Cu in the TSV layer increases (i.e., with a decrease in TSV pitch), the junction temperature still rises in the presence of the SiO_2_ layer. Only when the SiO_2_ layer is absent does the junction temperature exhibit a decreasing trend with higher Cu content. This behavior indicates that a more in-depth investigation into the influence of SiO_2_ on the equivalent thermal conductivity of the TSV layer is necessary.

**Figure 24:**
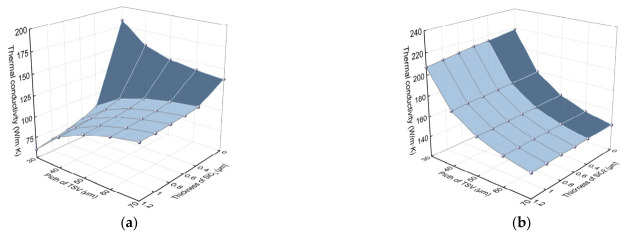
Equivalent thermal conductivity with different pitches of TSV and SiO_2_ thicknesses. (**a**) In-plane equivalent thermal conductivity; (**b**) out-of-plane equivalent thermal conductivity.

Due to its composition—primarily a silicon substrate with cylindrical vias filled with electroplated copper and a SiO_2_ layer of lower thermal conductivity—the thermal conductivity of the TSV layer exhibits marked anisotropy [26]; its in-plane and out-of-plane thermal properties differ. As shown in Figure 24, the in-plane effective thermal conductivity increases when the TSV pitch is enlarged or the SiO_2_ thickness is reduced. Because the thermal conductivity of SiO_2_ is significantly lower than those of Si and Cu, a higher SiO_2_ volume fraction naturally leads to a lower in-plane thermal conductivity. The SiO_2_ layer can therefore be regarded as a thermal barrier: as its volume fraction increases, the effective heat conduction paths through the Si are reduced, thereby suppressing the overall thermal conductivity. This trend is consistent with the observations reported in [8,27]. Although Figure 24b indicates that the out-of-plane thermal conductivity surges dramatically as the TSV pitch, the junction temperature of the 2.5D package still rises as the pitch is reduced. This suggests that the in-plane thermal conductivity of the TSV interposer plays a decisive role in the temperature distribution of the 2.5D package.

Furthermore, upon elimination of the SiO_2_ layer, the thermal conductivity increases markedly as the TSV pitch decreases, because the copper is directly incorporated into the heat conduction paths. This indicates that ignoring the SiO_2_ layer during homogenization would lead to an unrealistic and questionable estimate of the thermal conductivity. These results confirm that the SiO_2_ layer plays a critical role in the thermal behavior of the TSV interposer.

## 5. Discussions

This paper presents a numerical homogenization method based on PBCs, rigorously formulated to ensure strict satisfaction of the Hill–Mandel condition. By contrast, most current approaches employ an analysis based on material volume fractions to equivalently represent the complex multi-scale structures in 2.5D packages; these are commonly referred to as rules of mixtures. Although such analytical methods are computationally efficient, they are inherently approximate, and significant discrepancies exist among different mixture rule formulations, with no unified standard for their selection. Moreover, rules of mixtures are generally restricted to the homogenization of two materials, often neglecting the oxide layer when its volume fraction is small, as in TSV structures. However, the analysis presented in Section 4.2 demonstrates that neglecting the oxide layer is highly unreliable—simply ignoring its thermal conductivity can lead to substantially misleading conclusions. Reference [28] extended the rule of mixtures to three-phase materials by first homogenizing two materials and then applying the same rule to combine the resulting equivalent medium with a third phase. That study pointed out that the order in which materials are mixed affects the final equivalent result, which is physically inconsistent because the effective properties of a given composite structure should be unique. In contrast, the PBC-based numerical homogenization method proposed in this work enables the homogenization of thermomechanical properties for multiphase materials without being limited to two constituents, offering a more rigorous and physically consistent alternative.

Notwithstanding the methodological rigor established above, it should be acknowledged that the warpage and thermal predictions presented in Section 4.1 and Section 4.2 lack dedicated experimental validation for the specific 2.5D package design studied, as the fabrication and testing of the full package are planned as future work. Nevertheless, the proposed homogenization method itself has been rigorously validated against experimental data reported in the literature for equivalent elastic modulus [20], CTE/warpage [21], and thermal conductivity [22] (see Figure 10, Figure 11 and Figure 12 and the corresponding discussion in Section 3.3.2). These validations demonstrate that the method accurately predicts equivalent material properties with deviations of less than 5% from empirical measurements, providing strong confidence in its accuracy. The application to the specific package design presented in this study therefore serves as a demonstration of the method’s practical utility during the design phase, where rapid parametric studies are essential for design optimization. Experimental validation of the full package, including both warpage measurements (e.g., shadow moiré or profilometry) and thermal measurements (e.g., using thermal test chips or infrared thermography), is currently underway and will be reported in future work. Beyond the immediate application to thermo-mechanical analysis, the extensibility of the proposed method merits further discussion. Employing the PBC-based homogenization method to replace detailed heterogeneous structures with equivalent homogeneous models in FEA is expected to substantially reduce the mesh count, accelerate numerical convergence, and improve computational efficiency, as quantitatively demonstrated in Section 4.1.5. Moreover, since moisture diffusion and heat conduction are both governed by diffusion-type partial differential equations of analogous mathematical form, the PBC-based numerical homogenization method proposed in this work can be readily extended to the evaluation of equivalent moisture diffusion coefficients. Moisture diffusion analysis represents another critical challenge for 2.5D packaging technology, and the extensibility and transferability of the proposed method are therefore expected to provide valuable insights for such studies in 2.5D packages.

## 6. Conclusions

Due to the multi-scale nature of 2.5D packages, traditional FEA faces significant challenges in evaluating mechanical and thermal responses. Homogenization techniques that determine the equivalent material properties of multi-scale structures offer a promising alternative. This paper develops a PBC-based numerical homogenization framework and its corresponding implementation algorithm, combined with the rSVE concept, enabling the determination of the equivalent material properties of 2.5D package multi-scale structures within a unified algorithmic framework. The proposed PBC implementation has been validated to ensure the continuity of stress and strain at corresponding nodes on opposite surface pairs of rSVEs containing complex microstructures. Comparisons with data from existing studies further confirm the accuracy of the proposed homogenization method for the elastic properties, CTE, and thermal conductivity. The method achieves superior accuracy over traditional equivalent approaches, is not constrained by structural complexity, and remains efficient even for highly intricate multi-scale geometries.

The method has been applied to the thermomechanical analysis of 2.5D packages during manufacturing and service. It is found that incorporating the backside RDL reduces the maximum wafer warpage after debonding from 214.75 µm to 86.97 µm, demonstrating that the backside RDL plays a beneficial role in mitigating process-induced warpage. For the TSV layer, decreasing the Cu volume fraction reduces the maximum warpage in the x-direction from 93.50 µm to 73.59 µm, and in the y-direction from 105.43 µm to 86.82 µm. In contrast, increasing the SiO_2_ thickness raises the maximum warpage in the x-direction from 76.34 µm to 77.91 µm, and in the y-direction from 89.62 µm to 90.83 µm. Moreover, the effectiveness of the method in thermal analysis under the operating conditions of the 2.5D package has been demonstrated, and the influence of the SiO_2_ layer on the chip junction temperature has been investigated. Given its high computational efficiency, the proposed homogenization method is expected to play a valuable role in virtual manufacturing and the design optimization of advanced packages.

## Figures and Tables

**Table 1:** Linearly uncorrelated macroscopic fields are to be applied to rSVE for solving stiffness matrix ***C***.

Condition	$\varepsilon_{1}^{0}$	$\varepsilon_{2}^{0}$	$\varepsilon_{3}^{0}$	$\gamma_{4}^{0}$	$\gamma_{5}^{0}$	$\gamma_{6}^{0}$
1	$\varepsilon_{11}^{0}=1$	0	0	0	0	0
2	0	$\varepsilon_{22}^{0}=1$	0	0	0	0
3	0	0	$\varepsilon_{33}^{0}=1$	0	0	0
4	0	0	0	$\varepsilon_{23}^{0}+\varepsilon_{32}^{0}=1$	0	0
5	0	0	0	0	$\varepsilon_{13}^{0}+\varepsilon_{31}^{0}=1$	0
6	0	0	0	0	0	$\varepsilon_{12}^{0}+\varepsilon_{21}^{0}=1$

**Table 2:** Linearly uncorrelated macroscopic fields to be applied to rSVE for solving CTE.

Condition	$\varepsilon_{1}$	$\varepsilon_{2}$	$\varepsilon_{3}$	$\mu_{4}$	$\mu_{5}$	$\mu_{6}$
1	0	0	0	0	0	0

**Table 3:** Linearly uncorrelated macroscopic fields to be applied to rSVE for solving thermal conductivity.

**Condition**	$\nabla T_{1}^{0}$	$\nabla T_{2}^{0}$	$\nabla T_{3}^{0}$
1	1	0	0
2	0	1	0
3	0	0	1

**Table 4:** Various materials and their properties involved in the wafer warpage analysis.

Materials	Elastic Modulus (GPa)	Poisson Ratio	CTE (10^−6^ K^−1^)
PI	2.5	0.34	54 @25 °C
			50 @50 °C
			47 @100 °C
			35 @150 °C
			44.5 @200 °C
			122 @250 °C
Cu	117	0.34	17
Si	130	0.28	2.8
SiO_2_	73	0.17	0.5

**Table 5:** Each rSVE properties involved in the wafer warpage analysis.

Component	rSVE Size (µm)			Metal Volume Fraction	Material
	*L*_rSVE	*W*_rSVE	*H*_rSVE		
TSV layer	50	50	50	12.5%	Si, Cu, and SiO_2_
RDL1, 2, 3	100	100	10	40%	Cu and PI
RDL4, 5	25	25	5	61.44%	Cu and PI

**Table 6:** Mesh independence verification results for the rSVE of the RDL1 layer.

Mesh Size (μm)	Moduli (GPa)				Poisson Ratio		CTE (10^−6^ K^−1^) @25 °C		CPU Time (s)
	*E*_11_	*E*_22_	*G*_12_	*G*_23_	*ν*_12_	*ν*_23_	*α*_11_	*α*_22_	
4	47.38	8.757	2.841	1.620	0.06230	0.3372	18.16	40.80	2.95 × 10^1^
2	48.00	8.823	2.819	1.612	0.06248	0.3399	18.16	40.93	1.70 × 10^2^
1	48.22	8.811	2.816	1.611	0.06212	0.3399	18.15	40.94	2.53 × 10^3^
0.5	48.28	8.806	2.815	1.611	0.06201	0.3400	18.15	40.95	7.00 × 10^4^
Change (2 μm → 0.5 μm)	0.58%	0.19%	0.14%	0.06%	0.76%	0.03	0.06%	0.05%	/

**Table 7:** Equivalent material properties of each rSVE after numerical homogenization.

Component	Moduli (GPa)				Poisson Ratio		CTE (10^−6^ K^−1^)	
	*E*_11_	*E*_22_	*G*_12_	*G*_23_	*ν*_12_	*ν*_23_	*α*_11_	*α*_22_
TSV layer	127.90	158.91	72.17	49.32	0.29	0.11	4.72	4.83
RDL1, 2, 3	48.00	8.82	2.82	1.61	0.06	0.20	18.16 @25 °C	40.93 @25 °C
							18.04 @50 °C	38.34 @50 °C
							17.94 @100 °C	36.40 @100 °C
							17.57 @150 °C	28.64 @150 °C
							17.86 @200 °C	34.78 @200 °C
							20.30 @250 °C	84.91 @250 °C
RDL4, 5	72.54	15.50	5.28	2.84	0.07	0.15	17.49 @25 °C	30.81 @25 °C
							17.44 @50 °C	29.32 @50 °C
							17.40 @100 °C	28.20 @100 °C
							17.24 @150 °C	23.72 @150 °C
							17.36 @200 °C	27.27 @200 °C
							18.39 @250 °C	56.20 @250 °C

**Table 8:** Effect of Cu volume fraction in TSV layer on equivalent mechanical properties.

*P*_TSV_ (µm)	*V*_Cu_	Moduli (GPa)				Poisson Ratio		CTE (10^−6^ K^−1^)	
		*E*_11_	*E*_22_	*G*_12_	*G*_23_	*ν*_12_	*ν*_23_	*α*_11_	*α*_22_
30	8.73%	122.20	141.34	60.52652	46.26012	0.29950	0.17592	8.15	8.28
40	4.91%	126.12	153.18	67.16266	48.02282	0.29281	0.13726	5.80	5.95
50	3.14%	127.90	158.91	72.17197	49.31583	0.28745	0.10858	4.72	4.83
60	2.18%	128.85	162.14	74.36673	49.86676	0.28514	0.09581	4.13	4.22
70	1.60%	129.42	164.15	75.72121	50.19974	0.28376	0.08777	3.78	3.85

**Table 9:** Effect of SiO_2_ thickness in TSV layer on equivalent mechanical properties.

*T*_ox_ (µm)	*V*_SiO_2__	Moduli (GPa)				Poisson Ratio		CTE (10^−6^ K^−1^)	
		$E_{1}$	$E_{2}=E_{3}$	$G_{12}=G_{13}$	$G_{23}$	$v_{12}=v_{13}$	$v_{23}$	$\alpha_{1}$	$\alpha_{2}=\alpha_{3}$
0.4	0.52%	129.29	129.14	50.40	50.38	0.2811	0.2816	3.23	3.28
0.6	0.79%	129.13	128.90	50.33	50.30	0.2808	0.2812	3.23	3.27
0.8	1.09%	128.96	128.65	50.25	50.22	0.2804	0.2808	3.22	3.26
1.0	1.38%	128.78	128.40	50.17	50.13	0.2808	0.2804	3.22	3.26
1.2	1.69%	128.60	128.14	50.09	50.04	0.2795	0.2800	3.21	3.25

**Table 10:** Mesh convergence study for the homogenized wafer-level FEA model.

Mesh Density (Elements)	Max Warpage x-Direction (μm)	Max Warpage y-Direction (μm)	Change (%) Compared to Very Fine
45,000 (coarse)	91.23	94.56	~5%
180,000 (medium)	87.45	90.89	~0.5%
720,000 (fine)	86.97	90.52	<0.05%
1,440,000 (very fine)	86.94	90.48	/

**Table 11:** Various materials and their properties involved in the thermal analysis.

Materials	Thermal Conductivity (Wm^−1^K^−1^)
PI	0.8
Cu	386
Si	130
SiO_2_	1.5
SnAg	70.58
EMC	0.8
SnAgCu	58.7
Underfill	0.8
FR4	0.35

**Table 12:** Each rSVE property involved in the thermal analysis.

Component	rSVE Size (µm)			Metal Volume Fraction	Material
	*L*_rSVE	*W*_rSVE	*H*_rSVE		
TSV layer	50	50	50	3.14%	Si, Cu, and SiO_2_
RDL1, 2, 3	100	100	10	40%	Cu and PI
RDL4, 5	25	25	5	61.44%	Cu and PI
Micro bump layer	50	50	20	4.52%	SnAg and Underfill
C4 joint layer	500	500	160	0.46%	SnAgCu and Underfill

**Table 13:** Equivalent thermal conductivity of each rSVE after numerical homogenization.

Components	Thermal Conductivity (Wm^−1^K^−1^)	
	*k*_11_	*k*_22_
TSV layer	158.222	101.890
RDL1, 2, 3	151.497	3.665
RDL4, 5	235.201	6.430
Micro bump layer	9.147	1.097
C4 joint layer	5.239	0.977

## Data Availability

The original contributions presented in this study are included in the article. Further inquiries can be directed to the corresponding author.
